# Enhancing Healthcare Workers’ Capacity in Cancer and Other Non-Communicable Diseases (NCDs) Care in Sub-Saharan Africa (SSA) Through e-Health Innovations: A Case of International Cancer Institute (ICI), Eldoret, Kenya

**DOI:** 10.1200/GO.22.10000

**Published:** 2022-05-05

**Authors:** Gloria Kitur, Emmah Achieng, Carolyne Chemweno, Chite Asirwa

**Affiliations:** ^1^ International Cancer Institute, Eldoret, Kenya

## Abstract

Sub-Saharan Africa (SSA) hosts 11% of the world’s population yet bears 24% of the global disease burden with only 3% of the world’s health workforce at the frontline. The new threat of NCDs is worsened by the challenge of infrastructure, access to care and health workforce capacity. E-health bridges the gap of formal education and training through telemedicine and virtual e-learning platforms.

**METHODS:**

International Cancer Institute (ICI) leveraged on telemedicine to build capacity of Health Care Providers (HCPs) in the COVID era. Through bi-weekly virtual multi-disciplinary tumour boards, on-line preceptorship training programs and tele-consultations between healthcare providers across Kenya and SSA, access to quality and timely cancer care was evaluated.

**RESULTS:**

Between May 2020 and April 2021, ICI conducted 95 virtual multi-disciplinary tumour boards which were attended by 7,483 health care workers from 21 countries across the world, 16 from countries in SSA. ICI also conducted 27 virtual unique training programs in cancer and other NCDs care which attracted 578 participants from across the globe, 95% of which were from SSA. The training participants distribution included 77% clinical healthcare providers; 17% non-clinical health care workers and 6% allied healthcare workers. The online training platform provided the opportunity for HCPs to be trained from their locality thus bridging the gap of cost and distance to advance their training. The innovative e-learning platform has made it possible to reach more healthcare workers across SSA without the limitation of restricted classroom sessions.

**CONCLUSION:**

Capacity building of HCPs in SSA in cancer and other NCDs care and control goes beyond formal training. On-going peer to peer mentorship and on-job skills training have proved to be effective in building HCP capacity in improved standard of care across the cancer and other NCDs care. Use of a multi-disciplinary approach to care while leveraging on e-health solutions contributes to positive patient outcomes in resource limited settings.

